# Open radical prostatectomy reproducing robot-assisted radical prostatectomy: Involving antegrade nerve sparing and continuous anastomosis

**DOI:** 10.1590/S1677-5538.IBJU.2016.0627

**Published:** 2017

**Authors:** Se Yun Kwon, Jun Nyung Lee, Yun-Sok Ha, Seock Hwan Choi, Tae-Hwan Kim, Tae Gyun Kwon

**Affiliations:** 1 Department of Urology, Dongguk University College of Medicine, Gyeongju, Korea; 2 Department of Urology, Kyungpook National University Medical Center, Daegu, Korea

**Keywords:** Prostatectomy, Anastomosis, Surgical

## Abstract

**Purpose:**

To present modified RRP using the same method as RALP and compare its surgical outcomes with RALP.

**Materials and Methods:**

Demographics, perioperative and functional outcomes of the 322 patients that underwent RRP (N=99) or RALP (N=223) at our institution from January 2011 through June 2013 were evaluated retrospectively. Postoperative incontinence and erectile dysfunction are involved functional outcomes. During the modified procedure, the bladder neck was dissected first as for RALP. After dissection of vas deference and seminal vesicle, the prostate was dissected in an antegrade fashion with bilateral nerve saving. Finally, the urethra was cut at the prostate apex. After a Rocco suture was applied, and then urethrovesical anastomosis was performed with continuous suture as for RALP.

**Results:**

Perioperative characteristics and complication rates were similar in the RRP and RALP groups except for mean estimated blood loss (p<0.001) and operative time (p<0.001). Incontinence rates at 3 and 12 months after RRP decreased from 67.6% to 10.1 and after RALP decreased from 53.4% to 5.4%. Positive surgical margin rates were non-significantly different in the RRP and RALP groups (30.3% and 37.2%, respectively). Overall postoperative potency rate at 12 months was not significant different in RRP and RALP groups (34.3% and 43.0%).

**Conclusions:**

RRP reproducing RALP was found to have surgical outcomes comparable to RALP. This technique might be adopted by experienced urologic surgeons as a standard procedure.

## INTRODUCTION

Prostate cancer is the second most commonly diagnosed cancer and the sixth leading cause of male death worldwide (1, 2). Radical retropubic prostatectomy (RRP) has been established as a standard surgical treatment in patients with localized prostate cancer over several decades (3) after it was introduced by Walsh et al. (4). More recently, several surgeons have refined the surgical procedure and reported excellent outcomes (5, 6). However, RRP still presents technical difficulties due to a narrow surgical field and complex anatomy. In addition, the aim of RRP is to maintain oncologic principles and retain functional outcomes, which include urinary continence and erectile function.

Robot assisted laparoscopic radical prostatectomy (RALP) represents the leading application of robotic surgery in the urologic field. RALP has become the main treatment option for localized prostate cancer in worldwide and has been widely applied to improve operative outcomes (7–11). RALP involves advanced technologies that provide a 3-dimensional operative view, a laparoscopic instrument that mimics movements of the human wrist and hand, high-level resolution, enlarged images, and excellent lighting conditions. Therefore, RALP can be used to preserve neurovascular bundles more effectively and to enable the placement of anastomotic sutures in the narrow operative space without external loupes or a headlight. In addition, it has been reported that RALP is a feasible procedure that can enhance perioperative outcomes, by for example, reducing blood loss, hospital stays, and postoperative pain (12–14). Functional outcomes of RALP with respect to potency and continence have been evaluated in other studies and in terms of oncologic outcomes, RALP has replaced RRP for localized prostate cancer (15–17).

The widespread use of RALP has also contributed to the advancement of RRP. Numerous authors have compared the results of RALP and RRP in terms of surgical outcomes. However, the two techniques are quite different in terms of prostate dissection and urethrovesical anastomosis; RALP is conducted in an antegrade fashion using a continuous suture and RRP in a retrograde fashion using an interrupted suture. The widespread use of RALP has also contributed to advances of open prostatectomy. At our institution, we have been performing RRP using the method used for RALP, that is, using antegrade prostate dissection and urethrovesical anastomosis with a continuous suture. Here, we present the operative method of RRP and compare its pentafecta outcomes with those of RALP.

## MATERIALS AND METHODS

### Patients

We retrospectively analyzed the data of 322 consecutive patients that underwent RRP or RALP for prostate cancer between January 2011 and June 2013 (99 RRP and 223 RALP). This study was approved by the institutional review board and ethics committee of our hospital. The choice of surgical procedure was based on patient's demand and surgeon preference. Study participants were followed for at least 1 year. Institutional review board approval was obtained prior to data retrieval and analysis. All 322 patients underwent radical prostatectomy performed by a single experienced surgeon who had performed over 300 RALP procedures and 500 RRP procedures. Demographic data, operative parameters, pathologic data, postoperative complications, postoperative incontinence (PPI), erection function recovery rates of the two study groups were compared for pentafecta outcomes of radical prostatectomy.

### Surgical Technique

**Figure 1:**
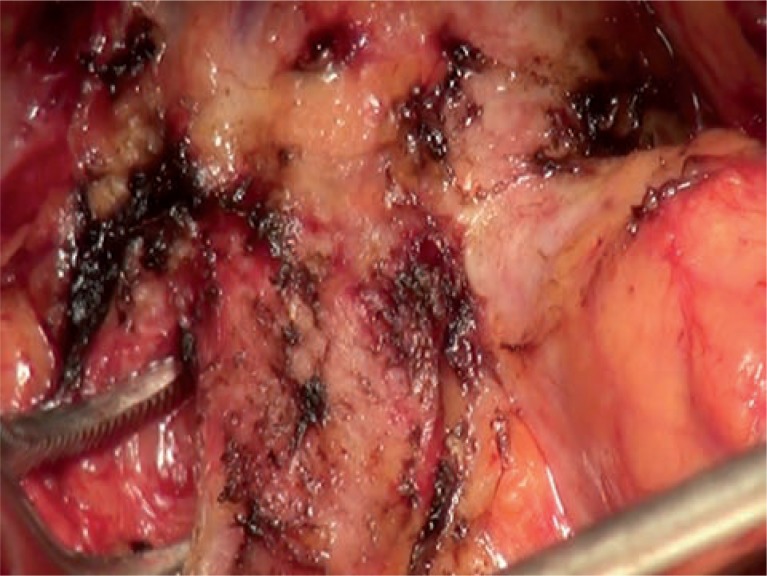
Lateral bladder neck dissection (radical retropubic prostatectomy). First dissection of both lateral sides of bladder and prostate borderline before anterior side dissection

**Figure 2:**
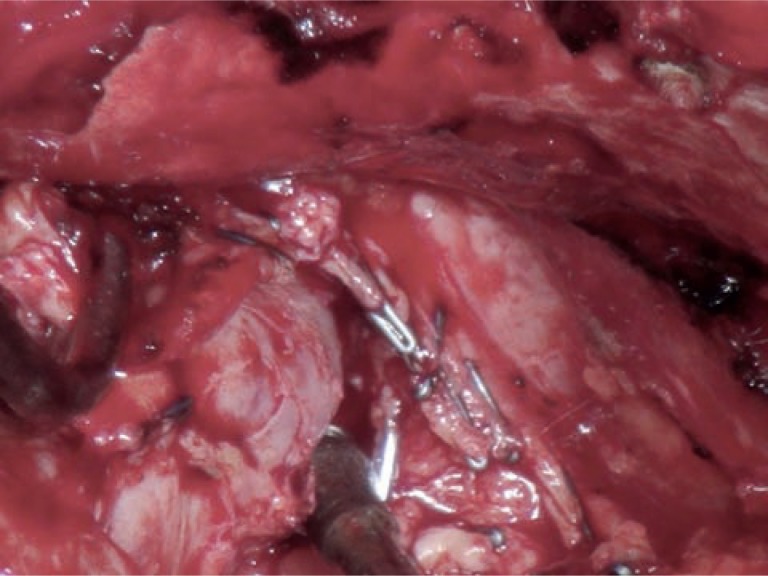
Antegrade prostate dissection including bilateral nerve sparing (radical retropubic prostatectomy). Antegrade nerve sparing of right lateral side

**Figure 3:**
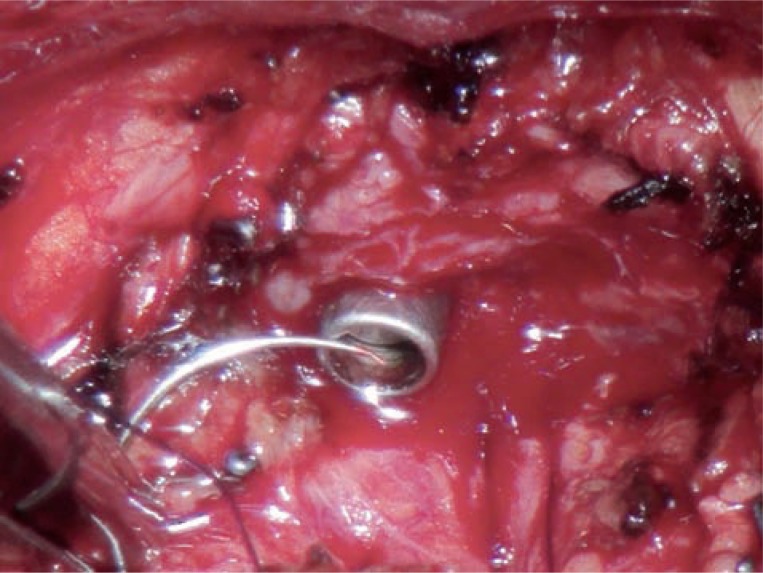
Continuous anastomosis (radical retropubic prostatectomy). First running suture directed from 6:00 to 3:00 o'clock

RALP was performed using a six-port transperitoneal approach using a four-arm da Vinci Si robotic system. In brief, patients were placed on the operating Table in the standard 30° Trendelenburg position. Laparoscopic adhenolysis was performed if required. The superficial dorsal vein was coagulated, divided, and preprostatic fat was removed. Both lateral sides of the bladder and prostate borderline were dissected first and then the bladder and prostate were divided using a Bovie knife along the bladder—prostate imaginary borderline until the prostatic urethra was exposed. The prostatic urethra was then incised and a previously placed Foley urinary catheter was observed, before continuing division of the bladder and prostate. When the prostate was completely divided, seminal vesicles and vas deferens were exposed and divided, vascular structures around them were ligated. Most vascular pedicles were ligated using 5mm Titanium Ligation clips (Aesculap, Melsungen, AG, Germany). Hem-o-lok clips (Teleex Medical, Durham, NC, USA) were used for large vessels unsuitable for ligation with titanium clips. Periprostatic tissue was then dissected antegrade fashion on each side by using scissors. Neurovascular bundles on both sides of the prostate gland were protected using an interfascial technique. The urethra was cut as distally as possible. The deep dorsal vein plexus was ligated by suturing after removing the prostate in an endo-pouch. After careful hemostasis, a Rocco suture was applied at the time of urethrovesical anastomosis. Posterior muscle-fascia was sutured parallel from the apical portion to the bladder neck using two 3.0 Monocyn^®^ (Aesculap, Melsungen, AG, Germany) strands tied together at their tails for the urethrovesical anastomosis. One strand of the running suture was directed right and the other directed left from 6:00 to 3:00 o'clock and from 6:00 to 9:00-o'clock, respectively. Anastomosis from 3:00 and 9:00 to 12:00 o'clock was performed while maintaining tension of the previous anastomosis using the third arm. At the end of this procedure a single tie was completed. The mucosa and serosa of the whole bladder neck were sutured divisively to prevent leakage and to tighten the anastomosis. A catheter was placed and the bladder was filled with 120mL of normal saline to check for leakage (Figures 1–3).

During RRP, patients were maintained in the standard 30° Trendelenburg position in common with RALP. The superficial dorsal vein and preprostatic fat were processed in the same manner as described for RALP. The bladder neck was dissected first after lateral bladder and prostate dissection and division at borderline as described for RALP. After dissection of vas deference and the seminal vesicle, the prostate was dissected in an antegrade fashion while preserving bilateral nerves. Finally, the urethra was cut at the prostate apex. After careful hemostasis, Rocco suture (3-0 monosyn) was applied at the same time as urethrovesical anastomosis. A two-strand running suture was placed in direction of both posterior sides first and then anastomosis was performed from 3:00 and 9:00 o'clock to 12:00-o'clock while maintaining tension at the posterior anastomosis site using a needle holder. A filling test was performed as described for RALP, and the Foley catheter used was removed on day 6 after surgery under cystographic control.

#### Definition and assessment of continence

Continence was defined as using no pads and having no urine leakages, as determined by patient responses. Patients were asked the following question: “How many pads or adult diapers did you use per day to control leakage during the past 4 weeks?”

Recovery of continence was evaluated routinely at 1, 3, 6, and 12 months after surgery. In addition, we compared the severity of incontinence in the two groups using 0-1 pad use per day.

#### Definition of erection function recovery

Erection function recovery was defined as the ability to achieve penetration ≥50% of the time and to maintain an erection significant enough for penetration ≥50% of the time as per questions 2 and 3 of the International Index of Erectile Function (IIEF)-5 survey at 12 months after surgery. Our most patients were prescribed PDE-5 inhibitors for 3 months except patients with contraindication.

#### Definition of biochemical recurrence

Biochemical recurrence (BCR) was defined as a serum PSA >0.2ng/mL on two consecutive measurements.

#### Follow-up evaluation

After hospital discharge, every patient was counseled to undergo a serum PSA test every 3 months for the first 2 years, every 6 months for the next 3 years, and then annually.

### Statistical Analysis

Demographics and perioperative outcomes were analyzed using the Chi-square test and the Mann-Whitney test. The Chi-square test was used to analyze incontinence rates and erectile function recovery rates at the above mentioned times. The Kaplan-Meier method and the log rank test were used to assess biochemical recurrence—free survival rates. The analysis was performed using PASW^®^ Statistics 18.0 (SPSS Inc., Chicago, IL, USA). For all comparisons, a p value of <0.05 was considered statistically significant.

## RESULTS

No significant differences were found between the RRP and RALP groups into demographic data, such as, age, body mass index, prostate volume, or preoperative PSA (prostate specific antigen). However, some operative parameters were found to be significantly different. In particular, mean estimated blood loss (EBL) was significantly higher in the RRP group (253.4mL vs. 192.6mL, p=0.001), but mean operative time was significantly shorter (188.8 min vs. 244.6 min, p=0.001). Pelvic lymph node dissection (PLND) was performed in 22 (22.2%) and 36 (16.1%) members of the RRP and RALP groups, respectively, and a nerve sparing (NS) procedure was performed in 65 (65.7%) and 177 (79.1%), respectively. Whereas this difference in PLND was not significant (p=0.190), the NS difference was significant (p <0.001). The urine leakage as determined by cystography was similar in the RRP and RALP groups. (13.1% vs. 9.0%, p=0.256) (Table-1).

**Table 1:** Demographic data & Operative parameter.

		RRP (n=99)	RALP (n=223)	p value
Age (years)		65.5±5.6	65.0±6.5	0.530
Body mass index (kg/m^2^)		23.6±2.5	24.0±2.8	0.243
Prostate volume (mL)		37.7±15.1	37.0±17.2	0.716
**Preoperative PSA (ng/mL)**				0.080
	< 10	53 (53.5)	136 (61.0)	
	10-20	25 (25.3)	61 (27.4)	
	> 20	21 (21.2)	26 (11.7)	
	PLND	22 (22.2)	36 (16.1)	0.190
	NS	65 (65.7)	177 (79.1)	0.001
Mean operative time (min)		188.8±62.3	244.6±60.0	0.001
Estimated blood loss (mL)		253.4±155.5	192.6±112.5	0.001
Urine leak on cystogram*		13/99 (13.1)	20/223 (9.0)	0.256

* At postop 7 days

**PLND =** pelvic lymph node dissection; **NS =** nerve sparing procedure

**Figure 4:**
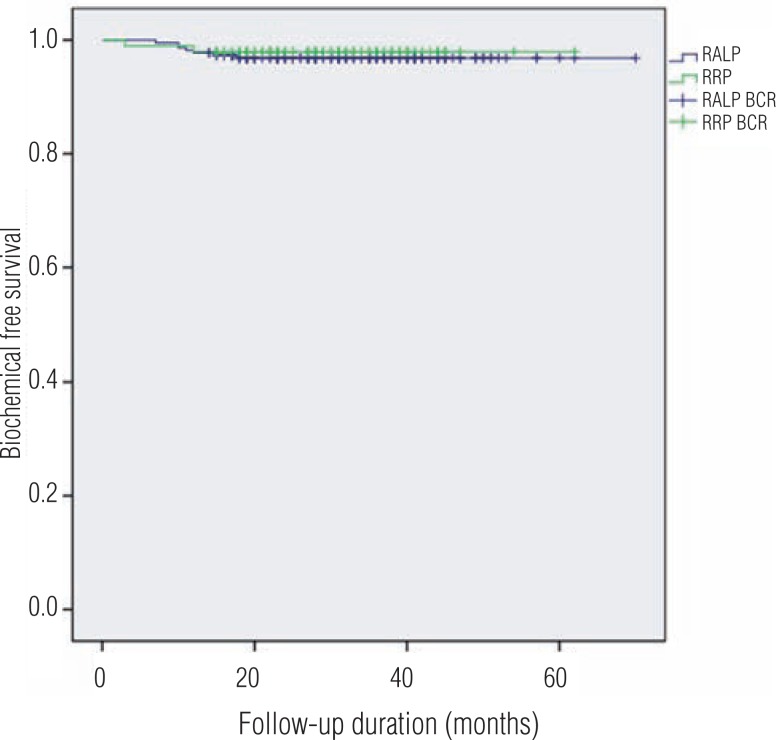
Overall 3-year biochemical recurrence-free survival rates.

For the 322 study subjects, mean and median follow-up were 31.4±10.3 months and 31 months (14–60), respectively. No intergroup difference was observed for pathologic stage or Gleason score. The positive surgical margin (PSM) rate was similar in the RRP group (30.3% vs. 37.2%, p=0.230). No intergroup difference was observed between PSM ranges (p=0.219). BCR occurred in 2 (2.0%) and 7 (3.2%) patients in the RRP and RALP groups, respectively. (p=0.574) (Table-2). Overall 3-year biochemical recurrence-free survival rates were 93.6% and 94.3% in the RRP and RALP groups (Figure-4).

**Table 2:** Pathologic data.

		RRP (n=99)	RALP (n=223)	p value
**Pathologic stage**				0.634
	T2	61(61.6)	129 (57.8)	
	T3a	26 (26.3)	65 (29.1)	
	T3b	12 (12.1)	26 (26.3)	
	T4	0 (0)	3 (1.3)	
**Pathologic Gleason score**				0.744
	6	33 (33.3)	86 (38.6)	
	7	37 (37.4)	80 (35.9)	
	8	24 (24.2)	42 (18.8)	
	9	4 (4.0)	13 (5.8)	
	10	1 (1.0)	2 (1.0)	
Positive surgical margin (%)		30/99 (30.3)	83/223 (37.2)	0.230
Focal/extensive		13/17	35/48	0.484
Biochemical recurrence (%)		7 (3.2)	2 (2.0)	0.574

Mean follow up duration: 31.4±10.3 months

Complication rates did not differ statistically in the RRP and RALP groups (5.1% vs. 2.7%, p=0.282). According to the Clavien classification (18), all complications were grade I or II and all cases were managed conservatively. In RRP group, 1 (1%) complication was grade I and 4 (4%) complications were grade II; in RALP group, 4 (1.8%) complications were grade I and 2 (0.9%) were grade II. One case of atelectasis, 2 cases of wound dehiscence and 2 cases of postoperative bleeding occurred in RRP group; 2 cases of atelectasis, 2 cases of ileus, 1 case of wound dehiscence and 1 case of pneumonia occurred in RALP group.

When continence was defined as no pad use per day, incontinence rates at 1, 3, 6, and 12 months decreased from 76.8% to 67.6%, 47.5%, and 10.1%, respectively, after RRP and from 70.0% to 53.4%, 39.5%, and 5.4% after RALP. The incontinence rate at 3 months was significantly higher in the RRP group (p=0.016), but excepting the third month, incontinence rates were similar during the 12-month follow-up period. When continence was defined as no pad or a single secure pad per day, the incontinence rates at 1, 3, 6, and 12 months decreased from 38.4% to 27.3%, 15.2%, and 4.0%, respectively after RRP to 22.9%, 16.1%, 9.4%, and 2.7% after RALP. Incontinence rates at 1 and 3 months were significantly higher in the RRP group.

With respect to erectile function, the overall postoperative potency rate at 12 months was 34.3% in the RRP group and 43.0% in the RALP group; however, this difference was no significant (p=0.142). Postoperative potency rates at 12 months were 32.3% and 43.1% in the RRP and RALP groups for nerve sparing procedure, which was not a significant difference (p=0.975).

The prescription rates at 3 months were similar in both groups (90.6% vs. 85.9%, p=0.209). However, prescription rates at 12 months were significantly higher in RALP group (63.6% vs. 74.4%, p=0.048).

The overall pentafecta rate at 3 months was 11.1% and 14.3% in the RRP and RALP groups respectively. The pentafecta rate at 12 months was 21.2% and 29.6% for each group respectively. Pentafecta rate in both was not a significant difference. The potency rate was most common reason for not achieving the pentafecta (Table-3).

**Table 3:** Pentafecta success rates between RRP and RALP at 6 and 12 months.

	3 months			12 months		
	RRP	RALP	p value	RRP	RALP	p value
No complication (%)	94.9	97.3	0.282	–	–	–
Negative PSM (%)	60.6	62.8	0.230	–	–	–
Continence (%)	32.4	40.6	0.016	89.9	94.6	0.121
Potent (%)	29.3	38.6	0.109	34.3	43.0	0.142
BCR (%)	1.0	0.0	0.133	2.0	2.1	0.900
Pentafecta (%)	11.1	14.3	0.430	21.2	29.6	0.118

**PSM =** Positive surgical margin; **BCR =** Biochemical recurrence; **RRP =** Retropubic radical prostatectomy; **RALP =** Robot assisted radical prostatectomy

## DISCUSSION

RRP is still the standard surgical treatment in terms of oncologic outcomes, but RRP is generally associated with significant decreases in quality of life as reflected by impotence and urinary incontinence rates. To address these problems, RALP has been widely introduced and has revolutionized prostate cancer surgery because of its associated magnified 3-D high-definition vision system and miniaturized wristed instruments, which allow microsurgery and respect of the most delicate anatomical structures. Furthermore, many recent technical and approach refinements during RALP have improved operative outcomes.

Different centers have reported widely varying comparative results for RRP and RALP. Rocco et al. compared the early oncological perioperative and functional outcomes of RALP (n=120) and RRP (n=240), and found RRP seems to be the faster procedure and that RALP provides better results in terms of estimated blood loss, hospitalization, and functional results, such as, postoperative incontinence and erectile function. Furthermore, early oncological outcomes appeared to be equivalent in their two groups (19). Ficarra et al. performed a non-randomized prospective comparative study of all patients that underwent RALP or RRP, and concluded RALP offers better results in terms of urinary continence and erectile function recovery with similar positive surgical margin rates (20). Krambeck et al. retrospectively analyzed data obtained from RRP (n=588) and RALP (n=294) procedures, and observed no significant intergroup difference for overall early complications, long-term continence, or potency rates. Furthermore, early oncological outcomes were similar in their groups (21). However, in most previously reports, RRP was performed by retrograde nerve sparing dissection with interrupted sutures, and RALP by antegrade nerve sparing dissection with a continuous suture.

Nerve sparing is an important step in radical prostatectomy and substantially determines functional outcomes, and hence, every attempt should be made to preserve neurovascular bundles (NVB). Two approaches to nerve sparing can be used, that is, from the prostate base to the apex (antegrade) or from the apex to the base (retrograde). During robot or pure laparoscopic surgery, the antegrade approach is mainly adopted because it is believed that it allows for early control of prostatic pedicles, and thus, minimizes bleeding during NS. Furthermore, this approach provides a more natural working angle for instruments during NVB dissection after the bladder neck has been divided.

Vesicourethral anastomosis (VUA) is another important step in radical prostatectomy, and has also been found to affect hospital outcomes. RRP is a modified version of the initial VUA technique described by Walsh et al., which makes use of interrupted sutures and is used in modern practice. However, interrupted suturing techniques are not used during RALP or laparoscopic radical prostatectomy (LRP) because of technical difficulties. Therefore, VUA using the continuous suturing technique introduced by Van Velthoven et al. (22) and modified by Menon et al. (23) is widely used. Several RALP and LRP studies using VUA and watertight continuous suturing have reported successful urethral catheter removal as early as 7 days after surgery (24, 25). In addition to its use in RALP and LRP, some studies have suggested that VUA with continuous suturing in open RRP could reduce VUA site leakage and alleviate PPI (26–28).

Before the introduction of RALP, we sutured the deep venous complex after opening endopelvic fascia, but this process created broad levator muscle injury, which is related to urinary incontinence. In addition, bleeding of pelvic muscles and adjacent tissues caused during this process can often obstruct the surgical field. These situations can be prevented by preserving the endopelvic fascia, and nerve-sparing procedures tend to be easier when the endopelvic fascia is preserved because it is not detached from muscle and the neurovascular bundle is relatively well—dissected. However, performing this technique was difficult in the narrow surgical field of RRP, which was adopted to prevent excessive bleeding. However, understanding of pelvic anatomy gained through experiences of robot surgery enable us to perform these ways. Furthermore, because the antegrade approach allows early control of prostatic pedicles, bleeding is minimized during NS and suturing of the deep dorsal vein complex is not required. Our VUA technique has several advantages. Because, we placed a Rocco suture and performed VUA simultaneously, posterior reconstruction approximated original anatomy. In particular, we sutured bladder mucosa and serosa separately, as a result, VUAs were watertight and in no patient was an anastomosis site torn intraoperatively. Furthermore, this procedure shortened the duration of indwelling Foley catheterization. In addition, anterior reconstruction was performed by suturing bladder serosa and prostatic fascia to include the prostatic ligament, which aided the recovery of continence and of the original anatomy.

The desirable results after radical prostatectomy include achieving oncologic and functional outcomes. Accordingly, trifecta or pentafecta represent optimal desired outcome and were used to help patients counseling undergoing radical prostatectomy. Antebi et al. reported trifecta following open radical prostatectomy. Trifecta at 2 and 5 years was achieved in 64% and 61% of patients respectively (29). Bianco et al. reported a trifecta rate of 60% at 2 years in 758 men after RRP (30). In our study, pentafecta rate at 12 months was 21.2% and 29.6% for each group respectively. Our results showed low rate relatively for previous other reports. It was considered that there were many high stage cases relatively in our cases for cited reports.

This study has some limitations that should be considered. First, no comparison was made between antegrade RRP and conventional RRP. Second, our results are based on a relatively small sample size because the study was performed using a retrospective design at a single institution. Nevertheless, the study shows that RRP can be improved by adopting what is essentially a RALP procedure. Moreover, our procedure can help to urologist trained with Robot system but affiliated in medical institution not equipped with Robot system.

## CONCLUSIONS

The surgical technique used during RALP, that is, antegrade dissection and continuous urethrovesical anastomosis, could be used for RRP. In the present study, antegrade RRP produced perioperative surgical outcomes comparable with that of RALP. We believe that this technique has the potential to be adopted by urologic surgeons as a standard RRP procedure.

## ABBREVIATIONS

RRP: = Radical retropubic prostatectomy
RALP: = Robot assisted laparoscopic radical prostatectomy
PPI: = postoperative incontinence
IIEF: = International Index of Erectile Function
PSM: = positive surgical margin
EBL: = estimated blood loss
VUA: = Vesicourethral anastomosis
PLND: = Pelvic lymph node dissection
NS: = Nerve sparing
PSA: = Prostate specific antigen
